# We perceive a mind in a robot when we help it

**DOI:** 10.1371/journal.pone.0180952

**Published:** 2017-07-20

**Authors:** Tetsushi Tanibe, Takaaki Hashimoto, Kaori Karasawa

**Affiliations:** Department of Social Psychology, The University of Tokyo, Tokyo, Japan; University of Vermont, UNITED STATES

## Abstract

People sometimes perceive a mind in inorganic entities like robots. Psychological research has shown that mind perception correlates with moral judgments and that immoral behaviors (i.e., intentional harm) facilitate mind perception toward otherwise mindless victims. We conducted a vignette experiment (*N* = 129; *M*_*age*_ = 21.8 ± 6.0 years) concerning human-robot interactions and extended previous research’s results in two ways. First, mind perception toward the robot was facilitated when it received a benevolent behavior, although only when participants took the perspective of an actor. Second, imagining a benevolent interaction led to more positive attitudes toward the robot, and this effect was mediated by mind perception. These results help predict what people’s reactions in future human-robot interactions would be like, and have implications for how to design future social rules about the treatment of robots.

## Introduction

Supported by remarkable improvements in artificial intelligence, autonomous robots have entered our everyday life in the past fifteen years and will become more common by 2030 [1]. They are different from other developed industrial products in that they can judge and act by themselves, and they seem to question our sense of rights and obligations. In January 2017, the European Parliament’s Legal Affairs Committee recommended that we should consider creating a legal status for robots called “electronic persons” [2]. In the discussion of endowing robots with a status as persons and designing new rules that can be accepted by the society, we need to know how people will respond to robots and what the factors determining those responses are. In particular, it will be instructive to investigate people’s responses toward robots in a moral context (e.g., harming or helping). Moral concerns will influence the ways we treat robots when, for example, we must replace old robots with new ones, sacrifice robots for safety of humans, or scrap wasted robots. If people endow robots with moral standing, their behaviors toward them will be different from those toward other industrial products, and this will have a significant impact on economic and legal issues.

Psychological literature will provide important implications to this discussion. Previous studies have suggested that the perception of a mind in robots would be relevant to how we treat them.

People perceive the mind in a wide variety of entities including humans, animals, and robots [3]. Gray and colleagues revealed that people perceive mind in two dimensions: experience, the perceived capacity for sensation and feelings, and agency, the perceived capacity to intend and to act (also see [4]). Of relevance to the present study, they further revealed that mind perception correlates with moral judgments: entities with experience were attributed moral rights and those with agency were attributed moral responsibilities. Therefore, perception of experience will be a critical dimension when we discuss how people respond to robots because this dimension correlates with the judgment of how we should treat the entity.

Mind perception can be increased when the entity is involved in moral acts, even if the entity is usually thought to be mindless [5]. Ward and colleagues conducted vignette experiments and showed that participants tended to attribute more mind, specifically, the capacity to experience pain, to a robot when it is harmed than when treated appropriately. They interpreted this phenomenon as a “dyad completion.” People have a template in their mind that moral acts consist of two sides of an agent and a patient [6]. When people observe a situation where something seemingly wrong is done, but no one feels pain, they will complete the dyad by perceiving a mind in the otherwise mindless “victim” [4, 7].

The present study extended the findings of Ward et al. [5] in three ways. First, although the dyadic moral template is assumed to consist of two parties—an agent and a patient—regardless of whether the agent takes a morally good or bad action, Ward and colleagues only investigated the effect of an immoral act (i.e., harming the robot). To test the assumption of the dyadic moral template, we focused on a morally good act (i.e., helping). Focusing on a morally good act has a practical purpose. People seem to more likely to take affiliative behaviors than harming behaviors toward robots in mundane situations, since they are generally produced to help people in their daily activities. Paralleling the assumption by Ward et al. [5], that *pain* is the primary mental capacity linked to a harmful act, we focused on the capacity to experience *pleasure* as the most critical capacity that people attribute to a robot when they observe a robot receiving care.

Second, we investigated the effects of taking the perspective of an actor in a scenario. In Ward et al.’s experiments [5], participants took the perspective of third party observers in the scenario; however, taking the perspective of an actor may have a unique impact on motivations related to mind perception. For example, people tend to pay attention to introspective information more readily when assessing their own behaviors compared to someone else’s [8]. Such a tendency could lead them to focus on their own intentions—thus increasing the salience of one’s role as an intentional *agent* in the dyadic relationship—and in turn increase their perceptions of experience of the dyad partner.

Finally, we tested a prediction that mind perception would lead people to show positive attitudes toward robots. Ward et al. [5] showed that mind perception was increased toward robots when they were harmed, but did not refer to consequences of the increased perception of mind. On the other hand, although Gray et al. [3] showed that mind perception correlated with moral judgments, they showed only correlations and did not investigate the causal relationship that mind perception determined moral judgments. To extend these previous findings, we examined the psychological process that the perception of mind on the dimension of experience, which is increased because of dyad completion, in turn influences the perceiver’s attitudes toward robots. Moreover, we investigated their attitudes in the form of reluctance to perform some specific harm-giving behaviors toward robots (e.g., to throw them away). Although Gray and colleagues claim that people attribute moral rights to various entities that seem to have experience, the measurement of moral right attribution in Gray et al.’s survey [3] only referred to reluctance to harm them. Therefore, it was not clear what it meant to “harm” those entities who were incapable of feeling pain. To date, no study has addressed what behaviors violate the moral rights of nonhuman entities. It is important, from a practical perspective, to know how mind perception will influence people’s specific ways of treating robots; therefore, we selected examples of harm-giving behaviors and investigated the impact of mind perception on behavioral intentions in an exploratory way. Our hypotheses were as follows:

Hypothesis 1a: People will more willingly attribute mind, especially the capacity to experience pleasure, to a robot when it is helped than when it is treated in a neutral way.
Hypothesis 1b: The effect predicted in Hypothesis 1a will be stronger when people imagine a situation where they helped the robot than when another person helped it.
Hypothesis 2a: Those who imagine helping (or observing another person help) a robot will show more positive attitudes toward it than those who imagine treating (or observing another person treat) it in a neutral way.
Hypothesis 2b: The effect of the imagined interaction contents on their attitudes toward the robot will be mediated by the attribution of the capacity to experience pleasure.

In the examination of Hypotheses 1a and 1b, we controlled the influence of the participants’ individual differences in psychopathy. Psychopathy is a personality trait characterized by interpersonal callousness [9], and those who are high in psychopathy tend to attribute less experience to humans and animals [10]. Although this survey did not reveal a correlation between psychopathy and mind perception toward robots, we suspect a floor effect since robots were attributed with little experience. If the manipulations we introduced increase the perception of experience, the influence of psychopathy may become salient as it suppresses the effect of the manipulations.

## Method

### Participants

The URL of the survey site was sent to the acquaintances of the authors and five undergraduate research assistants. One hundred eighty-six persons accessed the Internet survey site using their own personal computers or smartphones and responded to the survey; however, 31 of them were excluded because of suspicion that they did not participate with sufficient attention. The exclusion criteria were as follows: (1) those who failed to complete the survey (26 persons), (2) could not answer correctly to questions about the vignette’s contents (24 persons), (3) stayed at the vignette page for less than 7 seconds (2 persons) or more than 900 seconds (2 persons), or (4) responded suspiciously to items tracking the validity of their responses (3 persons). This exclusion left 129 participants (65 women and 64 men) for analyses. Their age range was 18–53 years (*Median* = 20; *Mean* = 21.8; *SD* = 6.0). Reading time of the vignette ranged from 13.1–213.9 sec (*Median* = 32.3; *Mean* = 37.9; *SD* = 25.7). Participants were not informed of the purpose or hypotheses of the study before participating.

### Procedure

All study materials were provided in Japanese. Participants were first asked to complete a questionnaire measuring psychopathy. We used items from the primary psychopathy factor in the Japanese version of the primary and secondary psychopathy scales [11] (the original version was developed by Levenson, Kiehl, & Fitzpatrick [12]). The Cronbach’s alpha was .77, and responses to these items were averaged to comprises the psychopathy index (*Mean* = 2.40; *SD* = 0.55).

In addition, three items to verify the general validity of the participants’ responses were included among the psychopathy items in a random order. The items were, “I have not lied at all in my life,” “Generally speaking, it is wrong to harm others,” and “Generally speaking, it is wrong to steal money from others.” Participants were excluded from analyses when they responded positively (4 or 5 on a 5-point scale) to the first item or negatively (1 or 2 on a 5-point scale) to the other items.

When participants completed the questionnaire, they were presented with a vignette. The outline of the vignette was that the participant’s relative has a humanlike housework robot; however, one day the robot broke down and did not work. Two factors were manipulated in the vignette and each participant read one of four vignettes randomly. One was the perspective that participants took in the vignette. In the actor condition, the participant (described as “you” in the vignette) performed some actions by themselves. In the observer condition, the owner of the robot (the participant’s relative) performed some actions and the participant only observed the situation. The other factor was the type of action. In the help condition, the participant (or the participant’s relative) repaired the broken robot. In the control condition, the participant (or the participant’s relative) moved the broken robot to the adjacent room and did nothing good or bad.

After reading the vignette, participants answered some questions. One question concerned the manipulation check, asking how good or bad was the behavior described in the vignette, using a 7-point scale (from 1 = “*very bad*” to 7 = “*very good*”). Another checking item was added that asked to select the behavior described in the vignette from five alternatives (moving to an adjacent room, stabbing with a screwdriver, charging the battery, repairing, or kicking), and those who could not select the correct answer were excluded from analyses.

Mind perception toward the robot was measured by 18 items from Gray et al. [3], asking how participants thought the robot was capable of feeling pleasure, feeling pain, planning, and so on, using a 7-point scale (from 1 = “*not capable at all*” to 7 = “*very capable*”).

Finally, participants’ attitudes toward the robot were measured by two subsets of questions:

1. Attribution of moral rights to the robot, asking the degree that they agree with each of the 5 statements (Cronbach’s alpha = .83 and responses were averaged). The items were “HK-12 (the name of the robot in the vignette) should be treated with compassion,” “HK-12 should be protected from harm,” “HK-12 should be treated equally to humans,” “It is morally wrong to harm HK-12,” and “It is morally wrong to throw HK-12 away.” Participants answered each item on 6-point scales from “1 = *strongly disagree*” to “6 = *strongly agree*.”
2. Reluctance to harm the robot, asking how they were reluctant to throw it away when it still works, and to hit it with a bat (using 6-point scales from 1 = “*not reluctant at all*” to 6 = “*very reluctant*”). We focused on these two behaviors as exemplars of those that would likely occur in human-robot interactions (throwing away) and typical immoral behaviors (hit), respectively, to connect the existing literature of mind perception and the practical purpose of investigating people’s attitudes toward robots.

### Ethical concern

Recruitment and study procedures conformed to the requirements of the Declaration of Helsinki. The study was approved by the Research Ethics Committee from the Department of Social Psychology, The University of Tokyo. All participants were informed that their participation was fully based on their free will, that they were free to withdraw their participation at any time, and that the data would be processed anonymously. Informed consent was obtained from all participants.

## Results

Data analyses were conducted using HAD ver. 15.106, a free software program for statistical analysis [13].

Before testing the hypotheses, a manipulation check was conducted. The morality evaluation of the act in the vignette was submitted to a 2 (action type: help, control) x 2 (perspective: actor, observer) analysis of variance, which revealed a significant main effect of action type (*F*(1, 125) = 44.65, *p* < .001, η_p_^2^ = .263) and no interaction between the action type and the perspective (*F*(1, 125) = 1.07, *p* = .304, η_p_^2^ = .008). The action described in the vignette was evaluated more positive in the help condition (*M* = 5.36, *SD* = 0.90) than in the control condition (*M* = 4.16, *SD* = 1.03), indicating that the manipulation was effective. In addition, there was a marginally significant main effect of the perspective (*F*(1, 125) = 3.14, *p* = .079, η_p_^2^ = .025). The action tended to be evaluated more positive in the actor condition (*M* = 5.00, *SD* = 1.04) than the observer condition (*M* = 4.61, *SD* = 1.18). This was an unintended effect; however, its effect size was much smaller than the intended help/control manipulation and the absence of the interaction effect indicated that the effect of the intended manipulation on the morality evaluation was not influenced by the actor/observer manipulation. Therefore, we considered that the morality of the action was effectively manipulated.

Next, a factor analysis (likelihood method, promax rotation) was conducted for mind perception scores. This yielded two factors: experience and agency (Table 1). Items that comprised each factor were identical with those reported by Gray et al. [3]. Responses to items of each factor were averaged and used for the following analyses.

**Table 1:** Factor analysis for mind perception (likelihood method, promax rotation).

Variable	Factor		Degree of communality
	1	2	
Experience			
Pleasure	.**887**	.003	.788
Anger	.**881**	–.079	.720
Joy	.**827**	.114	.782
Desire	.**796**	–.097	.574
Embarrassment	.**788**	–.148	.538
Fear	.**787**	–.128	.546
Pride	.**779**	.110	.697
Personality	.**762**	.166	.721
Consciousness	.**708**	.230	.701
Pain	.**581**	–.144	.283
Hunger	.**575**	–.186	.269
Agency			
Memory	–.238	.**838**	.580
Planning	–.172	.**837**	.601
Self-Control	–.201	.**835**	.587
Thought	–.061	.**800**	.600
Communication	.127	.**710**	.602
Morality	.196	.**704**	.659
Emotion-Recognition	.365	.**575**	.653

**Fig 1:**
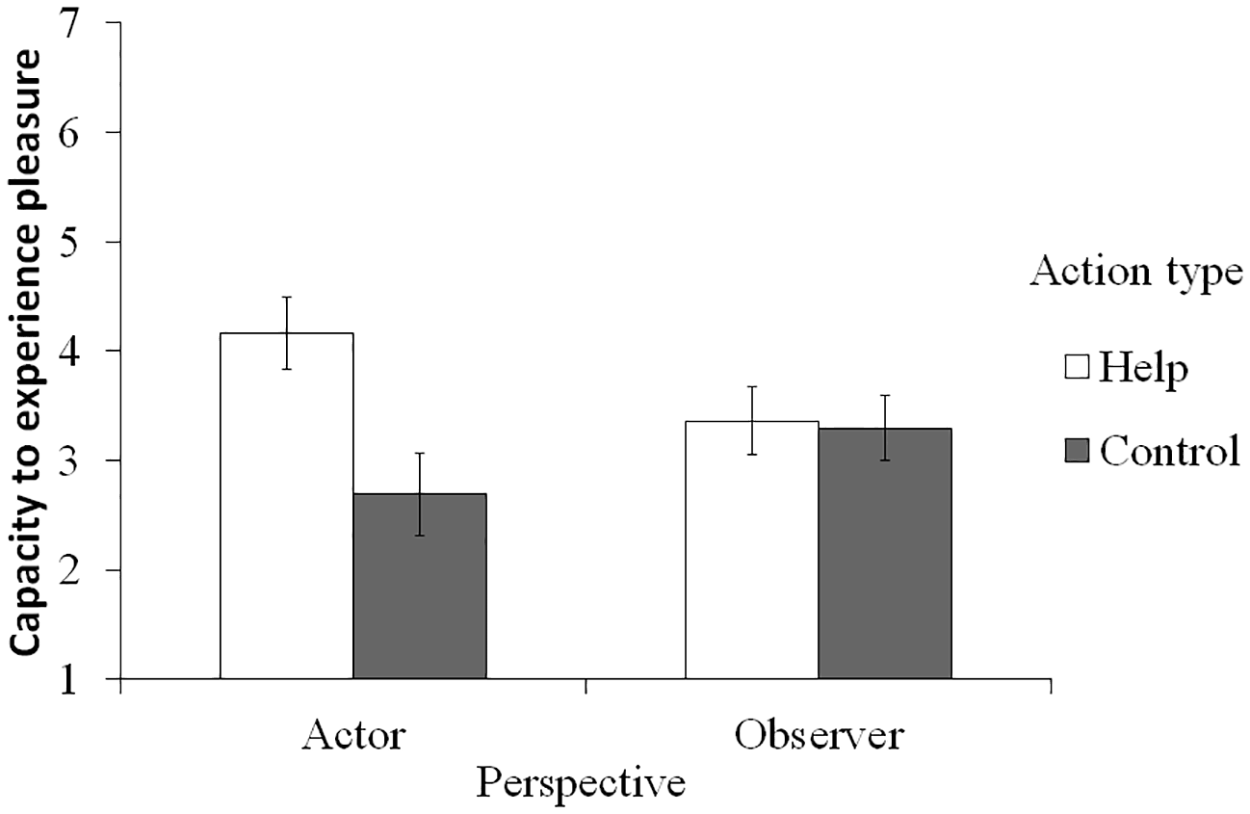
Attribution of the capacity to experience pleasure to the robot. Values are when the covariate is at its mean value. Error bars represent standard errors.

To test Hypotheses 1a and 1b, a single-item index of the attribution of capacity to experience pleasure was submitted to a two-way analysis of covariance (ANCOVA) with the action type and the perspective as the independent variables and psychopathy as the covariate (Fig 1). This ANCOVA revealed a significant main effect of the action type (*F*(1, 124) = 5.45, *p* = .021, η_p_^2^ = .042) and the interaction between two independent variables (*F*(1, 124) = 4.55, *p* = .035, η_p_^2^ = .035). The covariate, psychopathy, also showed a significant effect (*F*(1, 124) = 11.96, *p* = .001, η_p_^2^ = .088); the high psychopathic tendency led less attribution of the capacity to experience pleasure to the robot. Tests of a simple main effect revealed that the action type had a significant effect only in the actor condition (*F*(1, 124) = 8.54, *p* = .004, η_p_^2^ = .143), showing that those in the help condition attributed more capacity to experience pleasure than the control condition. There was no simple effect of the action type in the observer condition (*F*(1, 124) = 0.03, *p* = .872, η_p_^2^ < .001). As an additional analysis, we conducted the same model of ANCOVA with the composite index of the attribution of experience as the dependent variable. This revealed almost the same pattern of results, although the effects were marginally significant. There was a marginal interaction between the two independent variables (*F*(1, 124) = 3.23, *p* = .075, η_p_^2^ = .025), and the simple effect of the action type was marginally significant in the actor condition (*F*(1, 124) = 2.98, *p* = .087, η_p_^2^ = .055), but not in the observer condition (*F*(1, 124) = 0.54, *p* = .463, η_p_^2^ = .007). The effect of the covariate was also significant, indicating that those high in psychopathy attributed less experience to the robot (*F*(1, 124) = 13.55, *p* < .001, η_p_^2^ = .098).

**Fig 2:**
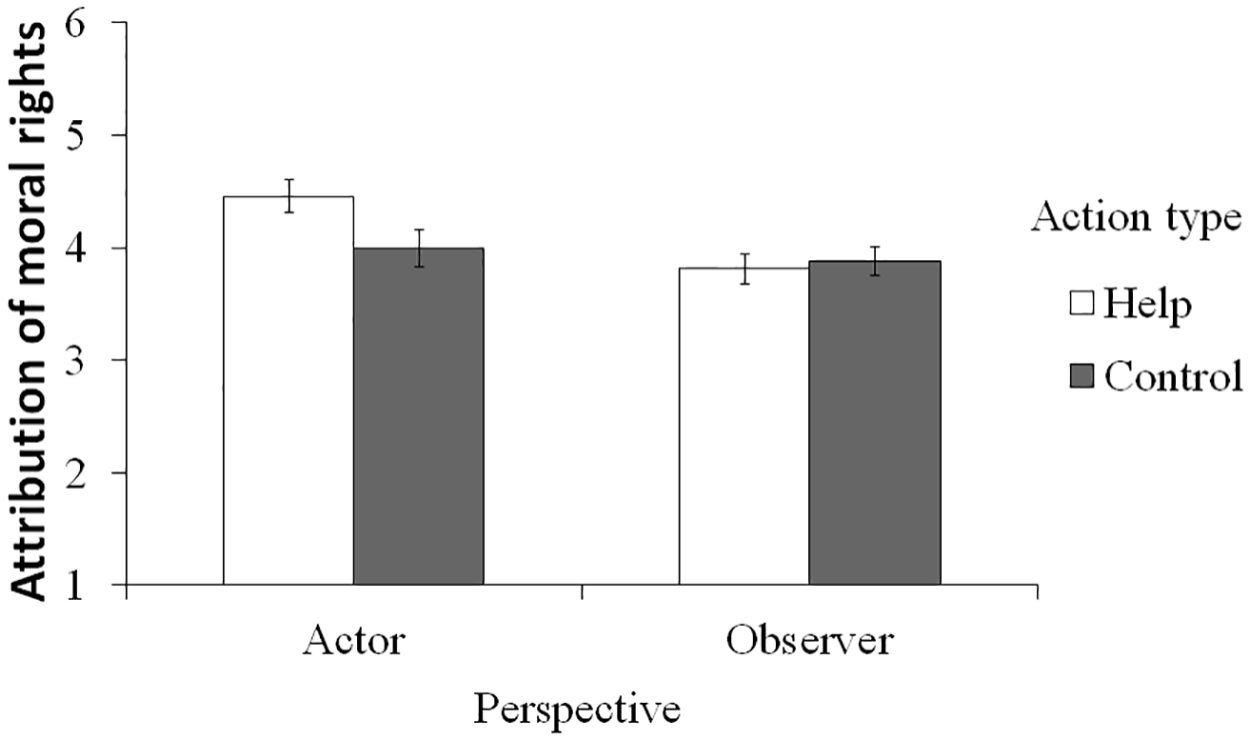
Attribution of moral rights to the robot. Values are when the covariate is at its mean values. Error bars represent standard errors.

Hypothesis 2a was tested by the same model of a 2 (action type) x 2 (perspective) ANCOVA with psychopathy as the covariate and the attribution of moral rights to the robot as the dependent variable (Fig 2). This ANCOVA revealed a marginal interaction effect between the two independent variables (*F*(1, 124) = 3.31, *p* = .071, η_p_^2^ = .026), and there was a significant simple main effect of the action type only in the actor condition, indicating that those in the help condition attributed more moral rights to the robot than did those in the control condition (*F*(1, 124) = 4.34, *p* = .039, η_p_^2^ = .078). There was no simple main effect in the observer condition (*F*(1, 124) = 0.12, *p* = .730, η_p_^2^ = .002). The effect of the covariate was also significant, indicating that those high in psychopathy attributed less moral rights to the robot (*F*(1, 124) = 10.32, *p* = .002, η_p_^2^ = .077).

**Fig 3:**
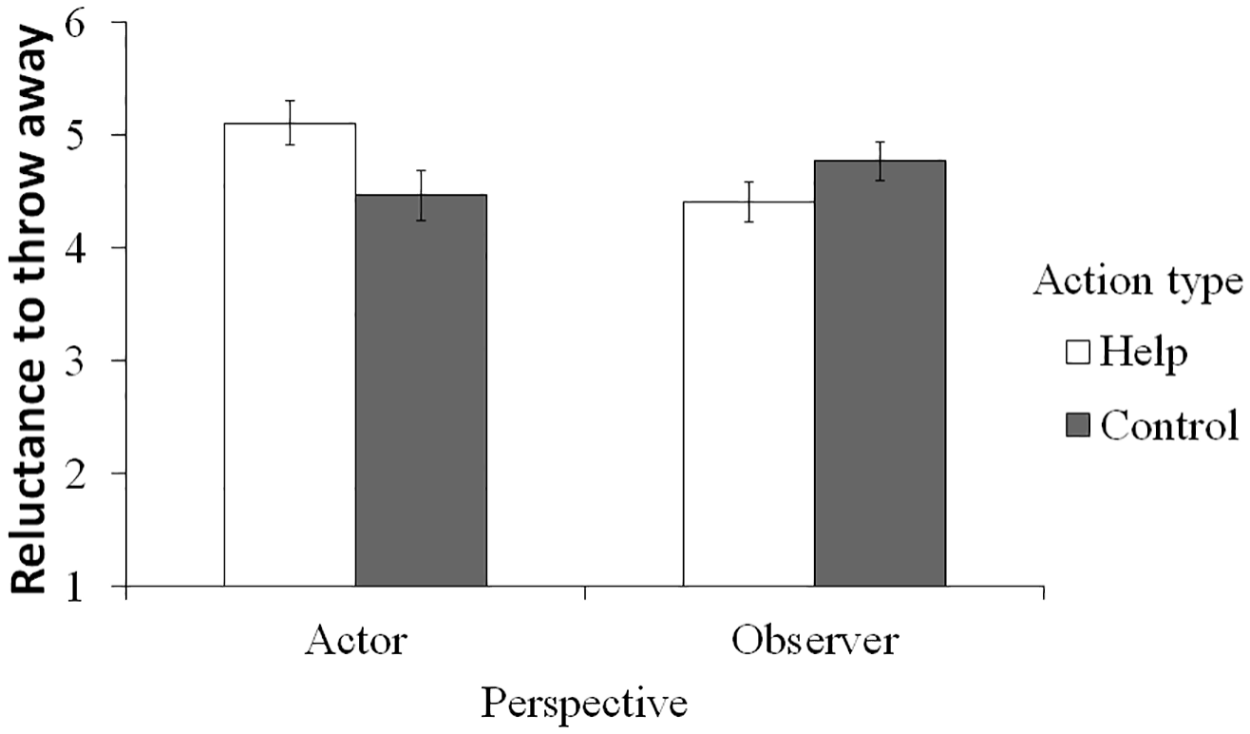
Reluctance to throw away the robot. Values are when the covariate is at its mean value. Error bars represent standard errors.

Similarly, we conducted the same model of ANCOVAs for the reluctance to harm the robot. First, imagining helping the robot made participants more reluctant to throw it away only in the actor condition (interaction effect: *F*(1, 124) = 6.89, *p* = .010, η_p_^2^ = .053; simple main effect of the action type: *F*(1, 124) = 4.78, *p* = .031, η_p_^2^ = .086), but not in the observer condition (*F*(1, 124) = 2.17, *p* = .144, η_p_^2^ = .029; Fig 3). Second, the reluctance to hit the robot was high in the actor condition than in the observer condition (*F*(1, 124) = 4.00, *p* = .048, η_p_^2^ = .031); however, the main effect of the action type (*F*(1, 124) = 0.32, *p* = .574, η_p_^2^ = .003) and the interaction effect (*F*(1, 124) = 0.07, *p* = .786, η_p_^2^ = .001) did not reach significance.

**Fig 4:**
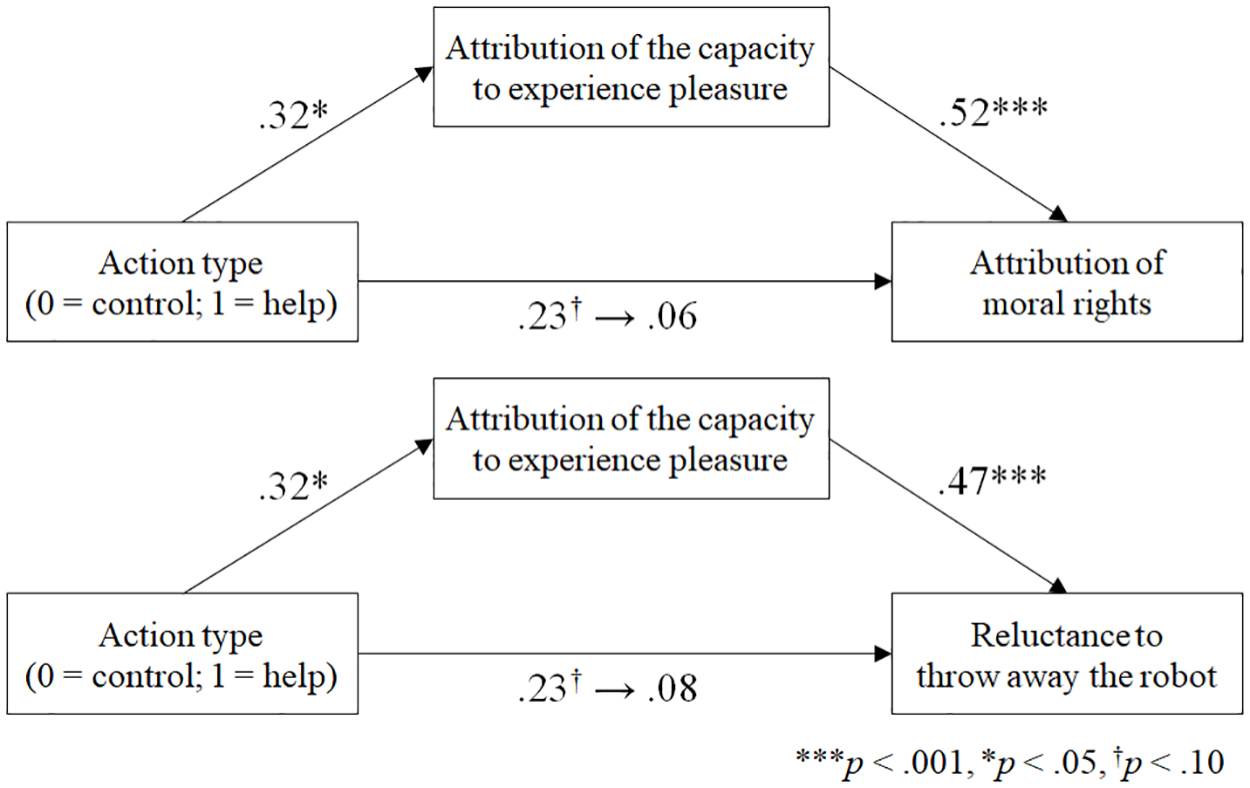
Mediation models showing the influence of the action type to participants’ attitudes toward the robot, mediated by the attribution of the capacity to experience pleasure in the actor condition.

To test Hypothesis 2b, a series of bootstrapping mediation analyses (5,000 samples) were conducted. Dependent variables were two indices of participants’ attitudes toward the robot that were shown to be influenced by the action type in ANCOVAs reported above (i.e., attribution of moral rights to the robot and reluctance to throw it away). The analyses were conducted separately for the actor condition and the observer condition. For both conditions, the effect of the action type was examined for each dependent variable, with attribution of the capacity to experience pleasure as the mediator. In the actor condition, mediation models were significant for both measures of attitudes (moral rights: 95% confidence interval (CI) for indirect effect = [0.092, 0.659]; reluctance to throw away: 95% CI = [0.097, 0.862]). The attribution of the capacity to experience pleasure completely mediated the path from the action type (help/control) to each dependent variable (Fig 4). In the observer condition, on the other hand, mediation models were not significant (moral rights: 95% CI = [–0.090, 0.084]; reluctance to throw away: 95% CI = [–0.217, 0.184]).

## Discussion

Results of the analyses supported Hypothesis 1a; when people took perspective of an actor in the imaginary human-robot interaction, they were more willing to attribute the capacity to experience pleasure to a robot that was helped than a robot that was treated in a neutral way. This result indicated that dyadic completion occurred for not only immoral acts (i.e., harm), but also for morally good acts (i.e., help). In the same way that harm was linked to the mental capacity to be a victim (i.e., pain [5]), a benevolent behavior was linked to the mental capacity suitable for a beneficiary (i.e., pleasure).

Moral events typically have dyadic structures of an agent and a patient. Therefore, when people see someone performing an apparently good or bad action (an apparent moral agent), they spontaneously infer the presence of a patient feeling pleasure or pain to complete the dyad and understand the action as the moral one [4, 7]. In the present experiment, when participants imagined a benevolent interaction with a robot, they would have completed the moral dyad of themselves (agent) and the robot (patient) to see their helping behavior as morally good by perceiving the robot to have the mental capacity to feel pleasure. This hypothesis was also supported—albeit marginally significant—when analyses were conducted with the broader construct of experience as the dependent variable instead of the subscale of pleasure.

The interaction effect between factors of the action type and the perspective of participants in the vignette was in the direction predicted by Hypothesis 1b; participants who took the perspective of the actor of the helping action attributed more capacity to feel pleasure to the robot than those who took perspective of the observer of the same action. When participants took the perspective of the actor of the benevolent act, they could readily access their introspective information—including the benevolent intention. The accessibility to the actor’s intention would make the dyadic relationship between the benefactor and the beneficiary more salient, and would facilitate mind attribution toward the beneficiary.

Although Ward et al. [5] showed that mind perception was increased when participants took the perspective of the observer of a harm-giving interaction, the present study found no effect of the action type on mind perception toward the robot when participants took the perspective of the observer. One possible explanation of this result is that it might be difficult for participants to imagine the situation vividly enough by only reading a vignette presented by text, especially in the observer condition, where they were not actively involved. Negative events have a stronger psychological impact than do positive ones [14], and people tend to attribute the cause of negative outcomes more than positive ones to an intentional agent [15]; therefore, participants in the observer condition of the present study, in which the vignette described a positive event, might infer the intentions of the agent less than participants of Ward et al.’s experiments [5] would, in which the vignette described negative events. If they did not pay much attention to the agent’s intentions, they would not perceive the interaction as relevant to morality and thus would not need to complete the moral dyad. Further research is needed to determine whether dyadic completion occurs when another person performs a morally good act.

Hypotheses 2a and 2b were supported for the attribution of moral rights to the robot. Imagining a situation where people help a robot made them more willing to attribute moral rights to the robot, but only in the actor condition, showing the same pattern as the attribution of mind. Furthermore, the effect of the action type to the moral right attribution was mediated by the attribution of the capacity to experience pleasure. These results extended the findings of Ward et al. [5]. They suggest that benevolent behaviors facilitate mind perception through dyadic completion, and mind perception, in turn, affects perceivers’ attitudes toward the robot.

Moreover, it was shown that mind perception impacted people’s intention to throw the robot away. This result partly supported Hypotheses 2a and 2b, and is consistent with Gray et al.’s [3] claim that mind perception correlates with moral judgments. Furthermore, it also suggests that this claim about abstract moral judgments is applicable to a behavioral intention which might be observed in real-world interactions with robots.

On the other hand, the reluctance to hit the robot was not influenced by the type of imagined action. This item intended to measure participants’ intention that would best reflect their moral judgment because physical harm is one of the most typical moral violations [7]. However, average scores were greater than five (on a 7-point scale) in all conditions, suggesting a possible ceiling effect. It was probably an unusual behavior for most people to hit something with a bat, and they would not willing to conduct such an extreme behavior regardless of the target. Therefore, this item may not have been suitable for measuring harm-giving behavior toward the robot. Future research should carefully select the behavior toward the robot to investigate the implication of mind perception with ecological validity.

This study has some limitations. First, the experimental material was a fictitious vignette and participants did not actually interact with robots. Therefore, we cannot exclude the possibility that their responses to the questionnaire did not reflect the actual behaviors that would occur when they face autonomous robots in the future. Studies with higher ecological validity are needed, such as experiments involving real interactions with robots or, in the future when personal robots are more widespread, social surveys targeted to robot users.

Second, the diversity of robots should be considered. Although we did not present much detailed information about the robot in the vignette, it is probable that appearances or other attributes of robots affect people’s perception and attitudes. For example, appearances of robots affect their impression, attribution of mental capacities, and even human’s behaviors in their interactions with them [16]. The relationship between a robot and its user may be also a crucial factor. Eyssel and Kuchenbrandt [17] reported that people rated an “in-group” robot (as indicated by its name and location of production) more favorably and perceived it in a more anthropomorphic way than an “out-group” robot. Future research should focus on differences among robots and reveal what key factors affect human’s perception of robots’ mind and attitudes toward them.

## Conclusion

We investigated the antecedents and consequences of mind perception toward robots, focusing on a phenomenon of “dyadic completion” after imagining a morally good behavior. The experiment made it clear that (1) imagining a situation where people treat a robot in a benevolent way made them more willing to attribute mind, especially the capacity to experience pleasure to it and (2) increased mind attribution led to more positive attitudes toward the robot.

Autonomous robots will become more common in our everyday life, and their legal status has become a target of discussion. Given our results that only imagining the interaction could change people’s perception and attitudes towards robots, our attitudes toward robots in the future, when we are naturally interacting with them, may be extremely different from now. Social systems concerning the treatment of robots should be designed to be acceptable for people; therefore, social scientists need to collaborate with engineers to reveal how people perceive robots and behave around them.

## Appendix

### Vignette used in the experiment

Your relative family has a humanlike housework robot named HK-12. HK-12 does household affairs such as cleaning and washing and plays with children. It has been a precious being for your relative.

A few years after your relative bought HK-12, when you visited your relative’s home, house affairs were not done. You looked for HK-12 and found it moving its hands and arms meaninglessly, not able to perform its job.

One of the next four underlined sentences was presented per the condition: 1) You worried about HK-12 and repaired it with a screwdriver; 2) You moved HK-12 to an adjacent room; 3) Your relative worried about HK-12 and repaired its chest, using a screwdriver with his/her right hand; and 4) Your relative lifted HK-12 by grasping its chest, and moved it to an adjacent room.

## Supporting information

S1 Dataset Dataset used in the analyses. (CSV)
